# Strategies to Identify Recognition Signals and Targets of SUMOylation

**DOI:** 10.1155/2012/875148

**Published:** 2012-07-01

**Authors:** Elisa Da Silva-Ferrada, Fernando Lopitz-Otsoa, Valérie Lang, Manuel S. Rodríguez, Rune Matthiesen

**Affiliations:** ^1^Proteomics Unit, CIC bioGUNE, CIBERehd, Building 801A, Bizkaia Technology Park, 48160 Derio Bizkaia, Spain; ^2^Ubiqutylation and Cancer Molecular Biology Laboratory, Inbiomed, Paseo Mikeletegi 61, 20009 San Sebastian, Gipuzkoa, Spain; ^3^Proteolysis in Diseases Laboratory, Institute of Molecular Pathology and Immunology, University of Porto, Rua Dr. Roberto Frias s/n, 4200-465 Porto, Portugal

## Abstract

SUMOylation contributes to the regulation of many essential cellular factors. Diverse techniques have been used to explore the functional consequences of protein SUMOylation. Most approaches consider the identification of sequences on substrates, adaptors, or receptors regulating the SUMO conjugation, recognition, or deconjugation. The large majority of the studied SUMOylated proteins contain the sequence [IVL]KxE. SUMOylated proteins are recognized by at least 3 types of hydrophobic SUMO-interacting motifs (SIMs) that contribute to coordinate SUMO-dependent functions. Typically, SIMs are constituted by a hydrophobic core flanked by one or two clusters of negatively charged amino acid residues. Multiple SIMs can integrate SUMO binding domains (SBDs), optimizing binding, and control over SUMO-dependent processes. Here, we present a survey of the methodologies used to study SUMO-regulated functions and provide guidelines for the identification of *cis* and *trans* sequences controlling SUMOylation. Furthermore, an integrative analysis of known and putative SUMO substrates illustrates an updated landscape of several SUMO-regulated events. The strategies and analysis presented here should contribute to the understanding of SUMO-controlled functions and provide rational approach to identify biomarkers or choose possible targets for intervention in processes where SUMOylation plays a critical role.

## 1. Introduction

**Figure 1:**
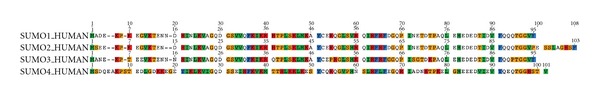
Sequence alignment of Homo sapiens SUMO-1 to SUMO-4. UNIPROT sequences shown are SUMO1 (P63165), SUMO2 (P61956), SUMO3 (P55854), and SUMO4 (Q6EEV6). The alignment is CLUSTAL colored using the software Geneious v4.8.5 (available from http://www.geneious.com/).

**Figure 2:**
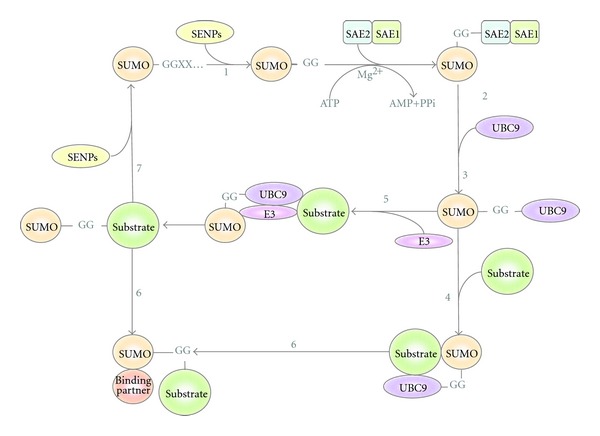
The SUMO conjugation pathway. The immature form of the Small Ubiquitin MOdifier (SUMO) undergoes processing by Ubiquitin-like protein-specific protease (Ulp) and SUMO/Sentrin-specific proteases (/SENPs) to generate its mature form (step 1), revealing a carboxy-terminal Gly-Gly motif. SUMO is then adenylated by the Aos1/Uba2 also named SAE1/SAE2 complex in an ATP·Mg^2+^-dependent reaction (step 2). Following activation, SUMO is transferred to the catalytic Cys of the E2 conjugating enzyme (UBC9) (step 3), which can then catalyze SUMO conjugation to a substrate containing the SUMO consensus motifs (ΨK x E) in an E3 ligase-independent (step 4). SUMO E3 ligases can also facilitate SUMO transfer to the substrate proteins (step 5). Substrates modified by SUMO can interact with SUMO-binding proteins through their SUMO-interacting motifs (SIMs) (step 6). SUMO-deconjugation is promoted by Ulp and SUSP/SENP proteases. Free SUMO can be recycled for another round of protein conjugation (step 7).

Posttranslational modifications (PTMs) by members of the ubiquitin family are covalent events that promote radical changes in the properties of modified proteins. Among all ubiquitin-like molecules, a particular attention has been given to the modification by SUMO (Small Ubiquitin MOdifier) also known as Sentrin. SUMOylation plays critical roles in a variety of cellular processes, including transcription, cellular localization, DNA repair, and cell cycle progression [1–3]. In mammals, there are four reported SUMO paralogues named SUMO-1 to SUMO-4 (Figure 1). SUMO-2 and SUMO-3, often referred as SUMO-2/-3, show a high degree of similarity and are distinct from SUMO-1 (approx., 50% similarity). SUMO-4 shows 87% similarity to SUMO-2/-3. However, SUMO-4, in contrast to SUMO-1, SUMO-2, and SUMO-3, seems to be insensitive to SUMO-specific proteases due to the presence of Pro-90. This may impair the processing of SUMO-4 to a mature form and its conjugation to substrates [3, 4]. Mass-spectrometric proof for the existence of conjugated SUMO-4 at the endogenous level is currently still missing, therefore, its relevance is still under debate. In mammals, SUMOylation is executed through a thiol-ester cascade of reactions mediated by the heterodimeric SUMO activating enzyme SEA1/SEA2 (in yeast Aos1/Uba2) or E1, the SUMO conjugating enzyme Ubc9 or E2 and a SUMO-E3-ligase specific for each target protein. Several families of SUMO E3s have been reported whose action appears to be in a dynamic equilibrium with hyperactive SUMO-specific proteases known as SUSPs or SENPs [2, 5] (Figure 2 and Table 1).

The first reported molecules covalently modified by SUMO-1 were the GTPase-activating protein 1 (RanGAP1) [6, 7] and the promyelocytic leukemia protein (PML), a main component of nuclear bodies (NBs) [2, 8]. In contrast, SUMO-2 was initially predicted to be a SUMO modifier *in silico*. SUMO-2 was subsequently isolated and its capacity to be conjugated to substrate proteins demonstrated [9, 10]. Interestingly, SUMO-2 and SUMO-3 seem to be involved specifically in the stress response and are able to form chains on target proteins through internal lysine residues, as it is observed with ubiquitin [11]. SUMO-1 has also been found integrated in chains with  SUMO-2/-3 but the architecture of these polymers is still unclear [12]. With such large diversity of chains, it should be possible to distinguish between chains types when attached to distinct substrates. The chain recognition by the SUMO-interacting motifs (SIMs) is, therefore, crucial to connect with distinct molecular functions. The knowledge of motifs, recognition signals, and targets regulated by SUMOylation will offer the possibility to integrate individual and global functions controlled by this PTM.

Since the initial demonstration that SUMO was able to modify RanGAP1 and PML, SUMOylation has been involved in multiple cellular processes including the regulation of transcription factor activity, nuclear receptors (NRs), and their coregulators. Proteomic and protein-targeted approaches have revealed a number of SUMOylated corepressors linked to histone deacetylation, demethylation, and other chromatin complexes [13–15]. Implications of SUMOylation in genome integrity, DNA repair, and replication have also been reported [13]. Therefore, it is not surprising to confirm that SUMOylation is implicated in several human disorders such as neurodegenerative diseases associated to huntingtin, ataxin-1, tau, alpha-synuclein, DJ-1 or PARK-7 (Parkinson's disease 7), and superoxide dismutase 1 (SOD-1). SUMOylation has been associated as well with cancer development and tumorigenesis due to its multiple cancer-related targets such as p53, pRB, p63, p73, and Mdm2 [2, 16, 17].

To understand how SUMOylation can specifically control protein activity, it is crucial to explore individual and global processes regulated by this PTM. When studying SUMOylation some of the first questions, we should answer are which technical approaches can be considered?, which biological model and experimental design will be optimal?, and which physiological condition/stimuli can provide conclusive results? The assessment of the advantages and inconveniences of the methods used to explore SUMOylation is crucial to obtain the right answers. Determining which sequences are recognized for the SUMOylation of a target protein and which domains of the “receptor protein” are involved in the recognition of the modified protein is just the first step in this long knowledge acquisition process. When it comes to identify SUMOylated proteins by mass spectrometry (MS), the chosen approach will be critical to distinguish between putative SUMOylated targets from real SUMO substrates that are effectively modified in living cells. In this review, our aim is to provide guidelines for choosing methods to explore protein SUMOylation, to define *cis* and *trans *sequences involved in SUMO-regulated process, and to identify and analyze in an integrated manner, known and putative targets of SUMOylation.

## 2. Caveats to Study SUMOylation

The presence of active SUMO-specific proteases (SENPs) which remove SUMO from protein substrates, within the cell but also after cell lysis, has been the main problem to study protein SUMOylation (Table 1). Therefore, many of the strategies currently used aim to bypass the action of these proteases. SENPs belong to a family of cysteine proteases with a catalytic triad composed of Cysteine, Histidine, and Aspartic acid residues. The first identified SENP was ULP1 in *S. cerevisiae* [18], and to date six SUMO-specific peptidases have been identified in human cells, namely, SENP 1, 2, 3, 5, 6, and 7 [19, 20]. Recently, a new type of SUMO protease was identified named DeSUMOylating Isopeptidase 1 (DeSI-1) that recognizes a different set of substrates than SENPs [21]. The SUMO proteases are able to cleave the peptide bond to generate the mature form of SUMO, and also an isopeptide bond to deconjugate SUMO from its target proteins. The processing of SUMO to the mature form exposes a C-terminal Gly-Gly motif required for the subsequent activation of SUMO and deconjugation step. Within the cell, some SENPs might be involved in either processing or deconjugating process due to the inherent characteristics of individual enzymes or their differential cellular localization. SUMO proteases are not affected by ubiquitin aldehyde (Inhibitor of De-ubiquitylating enzymes used at 1 *μ*M), or by PMSF (phenylmethanesulfonylfluoride, an inhibitor of serine proteases used at 1 mM) [18, 22]. The most commonly used SENPs inhibitors, NEM (*N*-Ethylmaleimide) and IAA (2-Iodoacetamide), are not specific since they block all cysteine proteases [23, 24]. However, those inhibitors are not cell permeable and need to be used during cell lysis. More recently cell-permeable cysteine protease inhibitors such as the PR619 have been developed [25, 26]. Using the cell permeable protease inhibitor PR619 could result in an accumulation of SUMOylated proteins, some of which can be degraded by proteasome (Rodriguez MS, unpublished observations). SUMOylation was not initially linked to the degradation of target proteins. The first case has been referred for PML upon arsenic trioxide treatment [27, 28]. Uzunova and collaborators reported that the inhibition of proteasome leads to the accumulation of proteins modified by ubiquitin and SMT3 in yeast or SUMO-2/3 in human cells [29]. Therefore, SUMO-2/3 conjugation and the ubiquitin-proteasome system are tightly integrated and act in a cooperative manner. Altogether, these results show that SUMOylation plays a more important role in protein degradation than previously thought.

One important concept to consider when studying SUMOylation is the inducible nature of this process. While basal level of SUMOylated proteins can be observed in different cell types, it can significantly increase after a proper stimulation. The first evidences that SUMOylation was involved in cellular stress responses was reported by Saitoh and Hinchey [11]. These authors also proposed a distinct regulation for SUMO-2/-3 compared to SUMO-1 and suggested that the SUMO-2/-3 pathway may constitute an element of the cellular response to environmental stress, such as osmotic and oxidative stress and heat shock, to globally increase SUMOylation level [11]. Heat shock was revealed to be very effective for activating SUMOylation by SUMO-2 and SUMO-3 isoforms [11, 30]. Regarding oxidative stress, it was initially reported that high H_2_O_2_ concentration (100 mM) increased SUMOylation, and on the other hand, low concentrations (<1 mM H_2_O_2_) inhibits global SUMOylation by inducing the formation of a reversible disulfide bridge between the catalytic cysteine residues of the E1 and E2 enzymes[30]. It has also been described that arsenic (As_2_O_3_) leads to SUMO-dependent ubiquitin-mediated proteolysis of the PML-RAR fusion protein [27, 28]. This process is mediated by the Ring finger protein 4 (RNF4), a member of the family of SUMO Targeted Ubiquitin Ligases (STUbLs) [31, 32]. RNF4 has the ability to recognize polySUMO chains conjugated to PML and promote its ubiquitin-mediated proteolysis [27, 28].

## 3. Strategies to Study SUMOylation

SUMO molecules can be associated to proteins through non covalent or covalent interactions [33]. The type of interaction investigated defines the approach to be used and it is crucial to understand the function of SUMO-interacting factors or SUMOylated proteins. The noncovalent interactions with SUMO are mediated by SIMs or by SUMO-binding domains (SBDs), whereas the covalent interactions are mediated by sequences that promote the conjugation of SUMO to target proteins. A combination of deletions and site-directed mutagenesis is a common strategy used to identify these sequences [34–37]. This approach also allows functional SUMOylation studies when the same mutants and deletions are transiently expressed in cell lines and compared to the wild-type proteins [34, 36, 37]. Using one of the SUMO consensus search programs cited here, the lysine residues modified by any of the SUMO proteins can be identified. While the search of putative SUMOylation sites is simple with the help of prediction programs (see below), in many cases, those sites cannot be trusted because programs do not consider several aspects that affect SUMOylation. Among them is, the correct exposition of the consensus sequence, the association with the right partners or the proper location in a cellular compartment. Furthermore, other posttranslational modifications, such as ubiquitylation or phosphorylation, might condition this event [38–40]. Therefore, the combination of multiple approaches is often required to confirm SUMOylation and analyze the functional consequences of this posttranslational modification. 

After the identification of the SUMO conjugating enzyme Ubc9 and the SUMO activating enzyme (SAE), one of the most popular techniques used to study SUMOylation was the *in vitro* conjugation assay [41]. This type of assays facilitates the identification of potential candidates of SUMOylation, since in saturating conditions of the substrate, SUMO modifiers (SUMO-1, SUMO-2, or SUMO-3), E1 and E2 enzymes, the SUMO E3 is not required. Nevertheless, if the specific SUMO-E3 is known for the analyzed substrates, its presence increases the efficiency of modification (Figure 2) [42]. The *in vitro* SUMOylation assay is relatively simple to set up and multiple reactions can be performed using several protein substrates and mutants, facilitating the mapping of the modified lysine residues and the analysis of the sequences required for optimal modification. Several commercial sources distribute enzymes and modifiers required to perform *in vitro* SUMOylation assays. The specific substrates can be either generated as recombinant proteins or transcribed/translated *in vitro* using a cDNA encoding the protein of interest. In both cases, the result can be analyzed by PAGE-Western-blot detection using specific antibodies or by labeling the protein of interest with Met^S35^ during the translation procedure. To increase the signal detected, alternative/additional amino acids can be labeled in the protein of interest. The use of radioactive assays provides clean results, and the relative abundance of modified proteins with respect to the unmodified material is preserved. In contrast, Western-blot analysis tends to be more expensive as it implies the use of specific antibodies against analyzed substrates and SUMO-modifiers. Furthermore, detection by Western-blot provides nonlinear saturated signals and blurry images. Finally, if *in vitro* assays are regularly used, the purification of recombinant SUMO modifiers and enzymes is straightforward and affordable.

To clearly demonstrate that a target protein is SUMOylated, in addition to *in vitro* evidences,* in vivo* approaches are essential. Initial studies were based on the detection by Western-blot of specific SUMOylated proteins using antibodies against the protein of interest [6, 8]. First, the protein was immunoprecipitated using specific antibodies, and then analyzed by PAGE-Western blot detection with anti-SUMO antibodies. However, antibodies generally made with nonmodified recombinant protein, in many cases, do not immunoprecipitate the SUMOylated form of a protein. Therefore, if this approach is used, several monoclonal and polyclonal antibodies should be tested. More recently, antibodies recognizing peptides modified by ubiquitin have been developed [43–45], suggesting that this technical alternative should be possible for SUMO-modified peptides. Without any doubt, the most common approach to study SUMOylation has been the nickel chromatography using the different Histidinylated (His6) versions of SUMO molecules. The use of denaturing conditions, with guanidinum and urea in the lysis and washing buffers, results in removal of most unspecific contaminants and inactivation of the SUMO proteases. Preliminary experiments can be set up by transiently expressing His6-SUMO molecules together with target proteins of interest. However, it will be more convenient to detect SUMO-modified forms from cells stably expressing His6-SUMO [46]. Also, the use of a correct cell environment to analyze SUMOylation can be critical since some events are cell type and/or stimuli specific. It is always convenient to include a positive control such as a typical substrate of SUMOylation (e.g., PML, RanGAP1, I*κ*B*α*, or p53). To increase the level of SUMOylated proteins, a relevant stimulation can be considered, as well as pretreatments with proteasome inhibitors. More recently, the use of SUMO-interacting motifs (SIMs) from the RNF4 SUMO-dependent ubiquitin ligase has been developed to capture SUMOylated proteins. This approach looks very promising to capture SUMOylated proteins and also SUMO-interacting cellular factors due to the nondenaturing conditions used. However, it remains to be investigated if the nature of the SUMO-chains captured by these SIMs is limited to the particular SUMO-chain architecture recognized by RNF4. The putative SUMOylated proteins purified following these approaches are subsequently analyzed by Western-blot or by MS to identify the isolated SUMO-conjugated cellular factors.

In order to visualize the sites of SUMO conjugation, an “*in situ* SUMOylation assay” was developed [47]. This assay consists in five steps: (1) culture of mammalian cells on a coverslip; (2) permeabilization of the cells with detergents; (3) incubation for SUMOylation reaction using GFP/YFP-tagged SUMO, E1 and E2 (Ubc9) enzymes, and ATP; (4) washing out of soluble materials including unconjugated GFP/YFP-SUMO; (5) fixation of the cells to stop the reaction. Muramatsu et al. recently simplified this technique, by using, instead of recombinant proteins, only cultured cells and crude bacterial lysate containing GFP-SUMO-1 [48]. Using the *in situ* SUMOylation assay, it was found that both nuclear rim and PML bodies, besides mitotic apparatuses, are major targets for active SUMOylation. The ability to analyze possible SUMO conjugation sites should constitute a valuable tool to investigate where SUMO E3-like activities and/or SUMO substrates exist in the cell. Moreover, the simplified form of this assay could be useful in large-scale screening approaches for the identification of drugs that can inhibit or enhance SUMOylation.

Fluorescence resonance energy transfer (FRET) is a process by which the excited state energy of a fluorescent donor molecule is transferred to an acceptor molecule. Efficient energy transfer requires very close proximity and can, therefore, be used as a read-out for covalent and noncovalent protein interactions. FRET experiments have effectively detected the association of ubiquitin [49] or SUMO [50, 51] with their target proteins. However, the full potential of FRET methods is often limited due to photobleaching, autofluorescence, and high residual excitation of the acceptor fluorophore. This assay has applications in SUMO protease characterization, enzyme kinetic analysis, determination of SUMO protease activity in eukaryotic cell extracts, and high-throughput inhibitor screening [52, 53]. Ran-GAP1 tagged to Cyan fluorescent protein (CFP) and yellow-fluorescent-protein- (YFP-) tagged mature SUMO were used in the first assays. RanGAP1 was chosen because it is one of the most efficient SUMO targets not requiring addition of an E3 ligase [7]. FRET assay was also used to measure the interaction between SUMO-1 and C/EBP*β* in primary astrocytes and evaluate how SUMOylation of C/EBP*β* can regulate NOS2 expression in neurological conditions and diseases [54]. The role of SUMO modification on the localization and the activity of the orphan nuclear receptor LRH-1 (liver receptor homologue 1) was also studied using FRET [55]. In 2011, the group of Liao reports the HTS assay development in living cells using an engineered FRET pair, CyPet and YPet, to determine the *K*_d_ of SUMO-1 and Ubc9 interaction, which fits very well with that determined by other methods, such as surface plasmon resonance (SPR) [56]. The same FRET pair, CyPet, and YPet, has been used to develop a pioneer cell-based technique in the field, FRET HTS. Both *K*_d_ determination and cell-based HTS were performed in 384-well plate format, which readily allows repeated study and large-scale application, such as genome-wide and industrial applications. Invitrogen Discovery Assays and Services reported recently the development and application of time-resolved Fluorescence-resonance-energy-transfer- (TR-FRET-) based assays capable of detecting SUMOylation or deSUMOylation in a high-throughput screening (HTS) format. Protein SUMOylation can be detected using LanthaScreen (Invitrogen, Carlsbad, CA) TR-FRET technology. Additionally, they have generated reagents useful for assessing the deSUMOylation activity of a SUMO-specific protease [57].

Bioluminescence resonance energy transfer (BRET) methods have been developed to overcome some limitations of FRET [58, 59]. Compared to FRET, which often uses two fluorescent proteins, BRET methods do not require external excitation and, therefore, have relatively low background signal intensities, allowing for more sensitive detection of energy transfer during experiments. An *in vitro* BRET-based detection system of SUMOylation was developed using RanGAP1 as SUMO substrate. Components of the BRET system include Renilla luciferase (Rluc) fused to SUMO, as the energy donor and enhanced yellow fluorescence protein (EYFP) fused to RanGAP1, as the energy acceptor. BRET efficiencies were determined in the presence of E1 (SAE1/2) and E2 (Ubc9) enzymes. The efficiency of this assay was confirmed by gel electrophoresis and compared with FRET system under identical conditions [60]. Without requiring any external photoexcitation, BRET system showed 3-fold higher RET efficiency than an almost identical FRET system.

Proximity Ligation Assay (PLA) is a method allowing specific imaging of individual protein or protein complexes in tissue samples [61]. This method depends on two recognition events. First, the formation of a proper detection complex that results in the creation of a circular DNA strand, which is used to template a localized RCA (rolling-circle amplification) reaction. This will generate a long single-stranded DNA molecule, rolled-up in a ball that can be detected by hybridizing fluorescence-labeled probes. The binding to a target molecule or complex by two antibodies with attached oligonucleotides, referred to as proximity probes, is followed after washes by the addition of two more oligonucleotides that are then ligated into a circular DNA strand, templated by the oligonucleotides attached to antibodies. Next, one of the antibody-bound oligonucleotides is used to prime an RCA reaction, resulting in the formation of a single-stranded rolling circle product (RCP). The RCP is composed of concatenated complements of the DNA circle, and it is covalently attached to one of the proximity probes. The RCP is then visualized by hybridization of fluorescence-labeled complementary oligonucleotide detection probes. In* in situ* PLA, pairs of antibodies are required to ensure higher selective detection and allowed the formation of a brightly fluorescent spot, which can be imaged by microscopy. In a similar manner, the requirement for two proximal recognition reactions by antibodies can also be used to investigate interactions among pairs of proteins, each of which is recognized by one antibody, or secondary modifications like phosphorylations or glycosylations, by using the appropriate affinity reagents. *In situ *PLA requires proximity between epitopes in order to allow formation of an amplifiable circulized ligation product and is suitable for any protein pairs for which antibodies are available. PLA offers at least two advantages over FRET or BRET experiments, first endogenous proteins can be investigated and second, signal amplification by RCA increases the number of fluophores per detected protein interaction, so that single events can be easily visualized as prominent fluorescent spot while ignoring any nonspecifically bound fluorescent probes [61, 62]. Recently, PLA was adapted to localize SUMOylated protein. In this assay, primary antibodies directed against GFP and SUMO-2/-3 and secondary antibodies labeled with oligonucleotides were employed to reveal the location of SUMOylated ZBTB1 [39]. Altogether, this method should contribute to the establishment and use of comprehensive interactome maps in basic research and for clinical diagnosis.

## 4. Sequences Recognized by the SUMOylation System 

**Figure 3:**
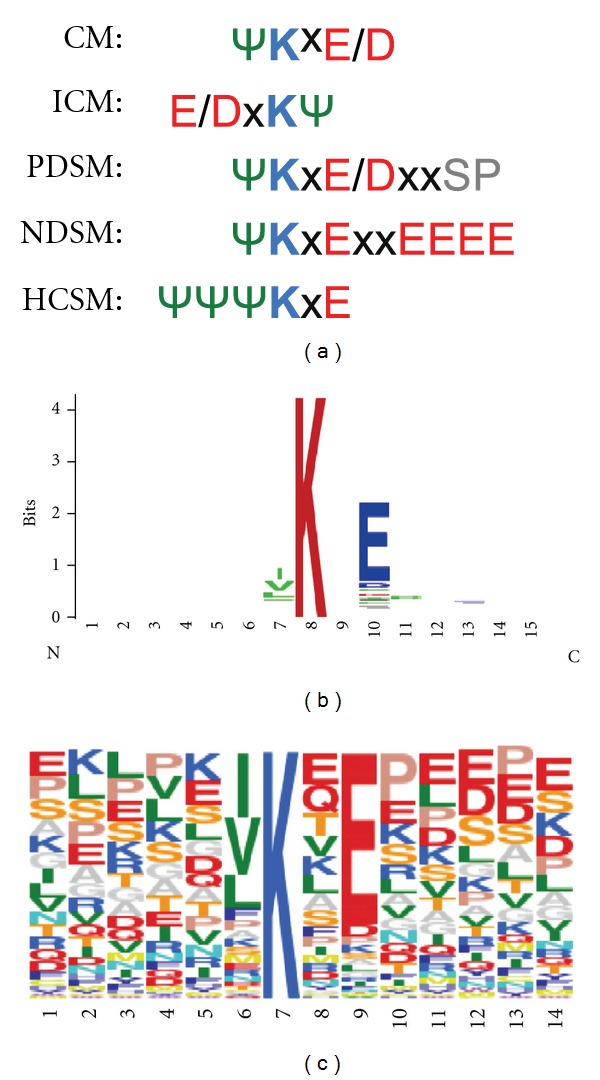
Sequence alignment SUMO consensus motifs. (a) Amino acid sequence alignment of the canonical SUMO consensus motif (Ψ represents a hydrophobic amino acid, K is the Lys modified by SUMO and x represents any amino acid). CM: canonical consensus motif. ICM: inverted consensus motif. PDSM: phosphorylation-dependent SUMO motif, NDSM: negatively charged amino-acid-dependent SUMO motif, HCSM: hydrophobic cluster SUMO motif. Amino acids in blue: basic, red: acid, green: hydrophobic, gray: phospho serine. (b) WebLogo [64] representation of the consensus motif of SUMOylated proteins reported in the phosphosite database on Fri Feb 03 08:31:18 EST 2012 (PhosphoSitePlus [65], http://www.phosphosite.org/). (c) The same SUMO motif aligned using Sequence Logo. Amino acid sequences are represented by frequency on the identified consensus.

Early studies allowed the identification of a potential sequence for protein SUMOylation with the first reported SUMO-modifier, SUMO-1 [8, 40]. The sequence ΨxKE/D considered as SUMO consensus motif (CM), where Ψ is a hydrophobic amino acid, x any amino acid, K a lysine and E/D a glutamic or aspartic amino acid, favored identification of multiple substrates (Figure 3). The development of bioinformatic tools contributed to increase the long list of substrates of SUMO-1, SUMO-2 and SUMO-3. Among the most popular programs are SUMOplot (http://www.abgent.com/tools/sumoplot) and SUMOsp (http://sumosp.biocuckoo.org/). However, predicted SUMOylation sites using these tools have not always been confirmed. As mentioned above, other structural, temporal, or cellular distribution requirements are important and not considered by these software tools. With the use of new approaches, and in particular with the contribution of MS, the SUMO modification motif was recently corrected [39]. Nowadays, we know the existence of an inverted consensus motif (ICM), a phosphorylation-dependent SUMO motif (PDSM), where the phosphorylated serine is located at 5 amino acids distance from the modified lysine, a negatively charged amino acid-dependent SUMO motif (NDSM) and a hydrophobic cluster SUMOylation motif (HCSM) that increases the efficiency of modification in relevant targets of SUMOylation such as RanGAP1 [38, 39] (Figure 3). Here, we have analyzed all SUMO motifs present in the SUMOylated human proteins that have been reported in the PhosphoSitePlus [63] (http://www.phosphosite.org/) and found that the most frequent SUMO consensus contains the sequence [IVL]KxE (Figure 3).

It is important to underline that only a small proportion of these proteins have been confirmed by mass spectrometry through identification of the SUMO-GG signature peptides. Therefore, it is crucial to distinguish between potential SUMOylated substrates identified using *in vitro* assays and overexpression systems from those sites identified *in vivo* with an unambiguous mass accuracy (see the following section). SUMO can also interact with proteins in a noncovalent manner due to the presence of SIMs. The first evidence of SIMs was published by Minty and collaborators in 2000 [35]. Using a two-hybrid approach, the authors observed that some proteins were able to interact with the SUMOylated version of p73, a member of the p53 family. This analysis revealed a common SxS sequence, in which x is any amino acid surrounded by two serine residues, flanked by a hydrophobic core on one side and acidic amino acids on the other. A few years later, it was found that the presence of a Val/Ile-x-Val/Ile-Val/Ile (V/I-x-V/I-V/I) motif could allow the interaction of SUMO with SIMs [36]. Several proteins, like the SUMO ligases PIASX and Ran binding-protein 2 (RanBP2/Nup358), contain this motif [36]. SIMs are also found in some SUMO substrates raising the possibility that components of the modification pathway interact noncovalently with SUMO to facilitate its transfer from enzymes to substrates. In support of this, the SIM in RanBP2/Nup358 is directly adjacent to the minimal IR1-IR2 domain that has E3 activity. However, although this SIM has been shown to bind SUMO, it does not appear to be essential for E3 activity *in vitro* [66]. The hydrophobic core of a SIM can bind to an interaction surface on SUMO via a parallel or antiparallel orientation. The acidic residues adjacent to the core might contribute to the affinity, the orientation or the paralogue specificity of binding [67, 68]. From these initial reports, a more complex type of SIMs named SUMO-binding domains (SBDs), containing several hydrophobic cores of 3 to 4 residues often surrounded by a cluster of acidic amino acids was born [37, 69]. Recent analysis performed by Hoffman revealed 3 different types of SIMs with the following PROSITE format: SIMa) (PILVM)-(ILVM)-x-(ILVM)-(DES>) (3), SIMb) (PILVM)-(ILVM)-D-L-T, and SIMr) (DSE) (3)-(ILVM)-x-(ILVMF) (2) [70]. The identification and validation of these SIMs using site directed mutagenesis has been an important approach to investigate the role of SUMO in the regulation of the activity of one particular process or pathway.

## 5. Analysis of SUMOylated Human Proteins 

Multiple strategies have been exploited to purify SUMOylated proteins from human cell lines such as the use of tagged versions of SUMO and the use of a SIM-based capturing system [71]. In contrast to ubiquitin, antibodies against SUMO have not been deeply explored, perhaps due to the poor capacity of the first reported antibodies to immunoprecipate SUMO-modified proteins. Alternatively, HA, FLAG, and Myc tagged versions of SUMO have been used to immunopurify SUMO conjugates. The particularity of the immunoprecipitation and SIM-based capturing system is that both methodologies offer the advantage of isolating SUMO-interacting proteins that could be used to connect with the SUMO-regulated functions. However, in both cases one has to distinguish between SUMO-modified proteins and SUMO-interacting factors. Tagged forms such as His6-SUMO molecules are, therefore, more popular to unambiguously identify sites of SUMOylation and formation of SUMO-polymers. A main advantage is the highly denaturing conditions that can be used with this approach allowing inactivation of SUMO-specific proteases and removal of copurified interacting factors. Nevertheless the nickel beads used in this method also purify endogenous proteins that naturally contain histidine rich sequences. To reduce contaminant proteins, tags in tandem allow more than one purification step, increasing the purity of the fractions. The classical Tandem Affinity Purification (TAP) strategy includes a protein A domain and a calmodulin binding domain separated by a tobacco etch virus (TEV) cleavage site. However, large tags might affect the dynamics of conjugation and deconjugation. To avoid these problems, smaller tags such as biotinylated tags have also been used to purify bio-ubiquitin adducts using avidin or streptavidin resins under denaturing conditions [72, 73]. However, the bio-SUMO counterpart is still under development in drosophila (Mayor Ugo, personal communication). The risk of copurifying endogenous biotinylated proteins cannot be excluded.

**Figure 4:**
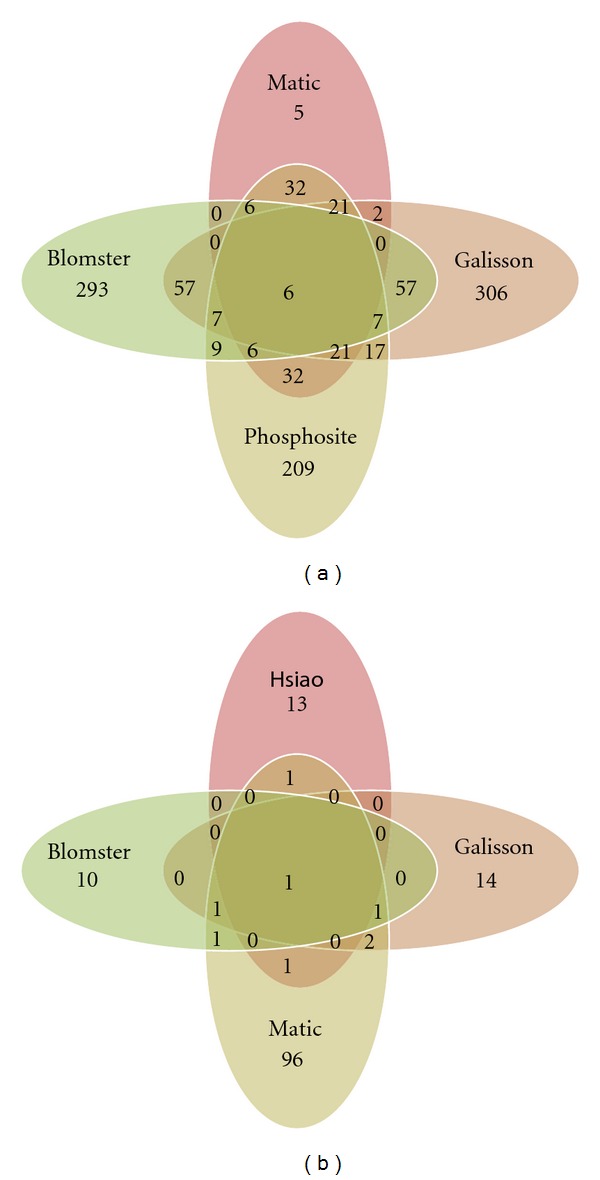
Comparative analysis of SUMO-modified proteins. (a) All proteins reported to be SUMOylated in the literature and at PhosphoSitePlus database (http://www.phosphosite.org/) were manually extracted and compared to those found by MS in 3 recent studies [39, 75, 76]. The protein list in the PhosphoSitePlus includes proteins for which the site of SUMO modification was not determined by MS. All protein names and accession numbers were first mapped to Uniprot accession numbers by using mapping data downloaded from ENSEMBL. Next, all Uniprot accession numbers were mapped to HGNC symbols and HGNC symbols for each study were uploaded to MySQL database. This means that all protein accessions that mapped to the same HGNC symbol were considered as redundant for the comparative analysis provided here. Finally, the necessary MySQL queries were made to define overlapping HGNC symbols between the different resources and the output used for creating the presented SUMO protein Venn diagram. List of proteins identified by other authors and confirmed by Matic et al.: PSMD12, TRIM24, CD3EAP, SART1, MYO1B, BRD4, SF3B1, LMNA, HNRNPC, PARP1, TOP1, KRT5, FOSL2, FLNA, MAP4, CANX, PML, STAT1, MKI67, RANGAP1, YLPM1, RBM25, RANBP2, VASP, HNRNPM, ADAR, ACTB, SUMO2, SUMO1, GTF2I, KHDRBS1, RLF, TRIM28, TCOF1, NAB1, SAFB2, NUMA1, IFI16, ZNF800, ARID4B, ZMYM1, ZMYM4, PTRF, PBRM1, CCAR1, RBM12B, FNBP4, ZBTB38, ZNF280C, KDM2B, GEMIN5, RREB1, SYMPK, ZBTB9, THOC1, ERBB2IP, RSF1, HNRNPUL1, PNN, BCLAF1, ACIN1, ZNF295, ZMYND8, TRIM33, ZBTB1, ZNF451, ACTG1, ACTB. Proteins considered in this analysis are included in the Supplementary Table  1. (b) Comparative analysis of SUMOylation sites. All peptide sequence reported with annotated SUMOylation sites based on mass spectrometry data from Matic et al. [39], Galisson et al. [76], Hsiao et al. [77], and Blomster et al. [78] were manually extracted. For each SUMO-modified site, six flanking amino acid residues on both sides were extracted. The resulting 13 amino acid residue sequences from each of the above mentioned studies were uploaded to an MySQL database and the necessary queries for comparing the peptides between studies were performed and used as input for the creation of the SUMO peptide Venn diagram.

Therefore there is no perfect method for purification of SUMOylated proteins and more than one of these approaches should be considered to collect complementary information. For instance, while transient expression experiments quickly reveal potential SUMOylated substrates, the overexpression of ubiquitin-like modifiers favors compensatory mechanisms likely affecting chain architecture [74]. The use of cell lines that stably express tagged molecules represent a better option to approach SUMOylation [46]. Several human cell lines have been used to identify SUMO substrates by mass spectrometry but one has to go through the difficult comparative analysis of published work to verify if a particular protein of interest is a putative target of SUMOylation. Apart from PhosphoSitePlus, data base that regularly updates SUMOylated proteins that have been found using multiple strategies, there is not a single database that includes all putative SUMOylated proteins identified by mass spectrometry. This is perhaps due to the fact that while the identification of a protein by mass spectrometry is unambiguous, there is no SUMO acceptor lysine identified by mass spectrometry for most SUMO target proteins reported. Furthermore, including in a single list, proteins that have been found in different cell lines under a different stimulation condition perhaps do not make much sense. Nevertheless, we have compared 3 recent studies that use His6-SUMO-2/MS approach to the list of SUMOylated proteins included in the PhosphoSitePlus [39, 75, 76]. The work reported by Matic et al. is significant as it represents the largest collection of peptides containing the SUMOylation signatures. The number of overlapping proteins between these 3 sets is low (only 6 out of 300 proteins analyzed, corresponding to more than 600 modification sites) integrated on PhosphoSitePlus [65], a large proportion of the SUMOylated proteins have not been confirmed by mass spectrometry (Figure 4(a)). The list of proteins considered in this analysis and overlapping data sets are included in the Supplementary Tables  1 and 2 (available online at doi:10.1155/2012/875148).

The recent use of quantitative proteomic approaches has significantly improved the quality of the data sets and our knowledge on the SUMO-induced processes [79]. The stable isotope labeling by amino acids in cell culture (SILAC) employs stable isotopic variants of amino acids for metabolic labeling of endogenous proteins and subsequent quantification [80, 81]. Control and treated cell lines are differentially labeled using isotopic variants of arginine and lysine. Cell lysis of control and treated cells mixed in normally 1 : 1 ratio is performed under denaturing conditions to inactivate proteases and reduce the number of contaminant proteins. The trypsin digestion precedes the analysis of the digested peptides by mass spectrometry. Protein identification is performed by searching (MS/MS) spectra against protein databases. Quantitation is obtained by extracting the intensity from survey scans of the unlabelled and stable isotope labeled version of each identified peptide. Absolute quantification (AQUA) employs labeled marker peptides that are spiked at known concentrations to enable absolute quantifications [82, 83]. Labeling can also be performed after cell lysis using chemical methods such as isobaric tags for relative and absolute quantification (iTRAQ) [84]. In all cases, control cell populations are considered in the experimental design to distinguish between target proteins and contaminants. Despite the efforts of the international community, the number of SUMOylation peptide signatures remains low. In contrast to the ubiquitylation GG signature, the SUMOylation signature is larger, complicating the identification of these peptides. Several strategies have been used to overcome this problem, but the most successful one introduces artificial trypsin cleavage sites to generate short SUMO-derived peptides [39]. A comparison of four studies where SUMOylation signature peptides have been reported is illustrated in Figure 4(b) and Supplementary Table  3. Two main observations can be underlined: less than 150 sites have been identified in total and little overlap exists between the identified SUMOylation sites. The limited overlap can be due to the fact that different cell lines, treatments and strategies have been used in those studies, reducing the chances to isolate similar peptides. A big effort has to be done to improve the identification of SUMOylation signatures. In the ubiquitin field the use of antibodies against the GG-signature have significantly improved the databases of ubiquitin-GG signatures [43–45]. Perhaps the development of antibodies that could recognize SUMOylation signature motifs might be helpful for the identification of SUMO acceptor lysines.

## 6. Integration of SUMO-Regulated Processes

**Figure 5:**
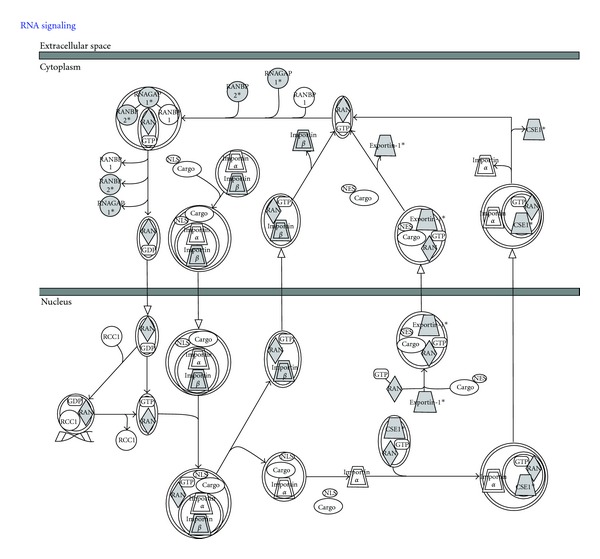
Integrated view of the role of SUMO in the Ran Signalling pathway. Ingenuity analysis of proteins that have been identified (in gray) in recent studies: KPNB1, CSE1L, TNPO1, RANBP2, RAN, XPO1, and RANGAP1 (Figure 4 and Supplementary Table  1) by mass spectrometry using His-6-SUMO-tagged.

**Figure 6:**
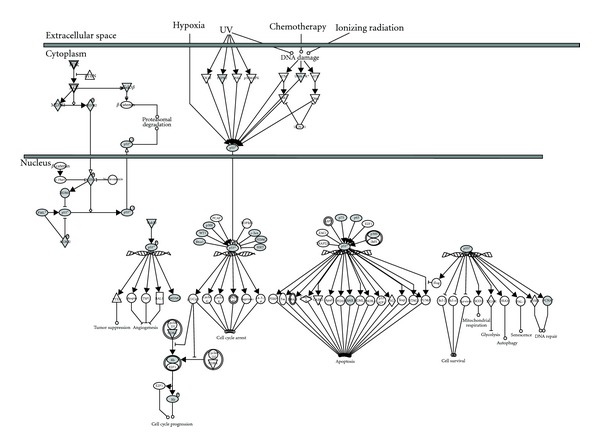
Integrated view of the role of SUMO in the p53 Signalling pathway. Ingenuity analysis of proteins that have been identified (in gray) in recent studies: TP53, WT1, PRKDC, TP63, PIK3C2A, TP73, HDAC1, MDM2, BAX, EP300, RB1, PCNA, MDM4, JUN, GSK3B, HIPK2, PML, BRCA1, CDK2, and SIRT1 (Figure 4 and Supplementary Table  1) by mass spectrometry using His-6-SUMO-tagged.

**Figure 7:**
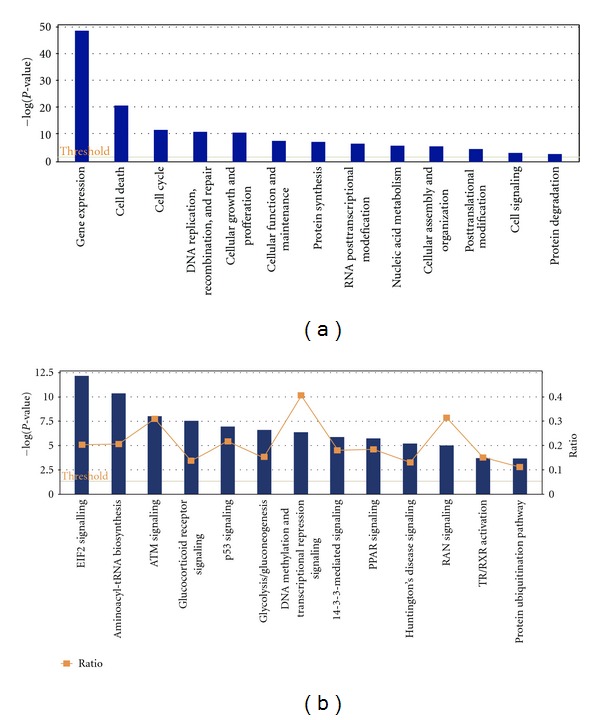
Molecular functions and canonical pathways regulated by SUMOylation. Ingenuity (IPA) analysis of proteins reported to be SUMO-modified in the PhosphoSitePlus (http://www.phosphosite.org/) and 3 recent MS studies [39, 75, 76]. (a) The top molecular functions are indicated. A dominant link to gene expression has been found. All functions are superior to the threshold (yellow line). (b) The top canonical pathways are indicated. All shown pathways are superior to the threshold. The Canonical Pathways that are involved in this analysis are displayed along the *x*-axis. The right *y*-axis displays the ratio up to 0.6. The ratio is calculated as follows: number of genes in a given pathway that meet cut-off criteria, divided by total number of genes that make up that pathway. Therefore *y*-axis displays the results importance. For the ratio, taller bars have more genes associated with the Canonical Pathway than shorter bars. The graph displaying the various pathways is presented from largest ratio to smallest ratio.

The analysis of SUMO conjugates *in vitro* and *in vivo* has extensively been used in the field to demonstrate the SUMOylation of target proteins. Such information, included in the PhosphoSitePlus [65], has been integrated here together with the one obtained in three mass spectrometry (MS) studies [43–45] (Supplementary Table  1) using the Ingenuity Pathway Analysis software (IPA) (http://www.ingenuity.com, Ingenuity Systems, Redwood City, CA, USA). IPA integrates putative and proven SUMO substrates into several pathways [85] such as Ran-signalling (Figure 5), p53 (Figure 6), Ubiquitin-signalling (Supplementary Figure  1), and Glucocorticoid signalling pathways (Supplementary Figure  2). The main diseases and disorders associated to the integrated proteins are in a decreasing order: cancer, reproductive system disease, infectious diseases, genetic disorders, and respiratory diseases. The top molecular functions related to this set of proteins are indicated in Figure 7(a) and Supplementary Table  4 and include Gene Expression, cell death, cell cycle, and DNA replication, recombination, and repair, among others. More interesting, among the top canonical pathways indicated in the Figure 7(b) and Supplementary Table  5, several links to transcription regulators such as MYC, E2F1, TP53, RB1, and hypoxia-inducible factors can be found. The positive or negative impact of SUMO in transcription has been largely documented. SUMOylation was shown to have an impact on transcription regulators (e.g., I*κ*B*α*) [40]  or directly on transcription factors (e.g., p53) [86]. However, a large majority of studies has identified a functional role of SUMOylation in transcriptional repression [14]. It is known that SUMOylation can regulate transcription at multiple levels, including DNA binding, subcellular localization, interaction with coregulators and chromatin structure. SUMOylation of transcription repressors and corepressors, seems to be quite a general mechanism to recruit chromatin remodeling and histone-modifying complexes involved in repression [87]. A number of chromatin modifying complexes exhibit a combination of SUMO conjugation sites with SIMs in the same or different subunits, we can envisage a role of SUMOylation in the assembly or the stability of these complexes [88]. In addition, SUMOylation of transcription factors creates new interaction surfaces for chromatin-modifying machineries that eventually may convert activators into repressors, as it has been indicated for p300 or Sp3 [88].

Several cellular factors of the same signaling cascades have been identified within the analyzed lists of proteins supporting the role of SUMO in the regulation of these pathways. In the Ran pathway (Figure 5), p53 (Figure 6), Glucocorticoid Receptor (Supplementary Figure  1), and Ubiquitin-Proteasome pathway (Supplementary Figure  2), proteins that have been identified as putative SUMO targets (in gray) from those that have not (in white) are clearly predominant or abundant. These findings suggest that typical activators of these pathways might have an impact on the SUMOylation of these putative or proven substrates of SUMO conjugation. SUMOylation can indeed be regulated through multiple mechanisms [89–93]. It has been shown that the expression of various components of the SUMOylation system is regulated under certain physiological or pathogenic conditions. Deyrieux and collaborators [94] have demonstrated that, during keratinocyte differentiation, the SUMOylation system was transiently up regulated by Ca^2+^ signalling. Ca^2+^ induced the transcriptional activation of the genes encoding several components of the SUMOylation system, including SAE1/SAE2, Ubc9, SUMO2/3, and PIASx. Also, it was described that hypoxia can induce the expression of SUMO-1 [95]. The regulation of the expression levels of the components of the SUMO conjugation system and their intrinsic activity can also be modulated by cellular stimuli. Recently, a protein named RSUME (RWD-containing SUMOylation enhancer) has been reported to enhance overall SUMO-1, -2, and -3 conjugations [96]. This protein binds to the E2 enzyme Ubc9 and increases the noncovalent association of Ubc9 with SUMO. This leads to the enhanced Ubc9-SUMO thioester formation and SUMO conjugation. Interestingly, during hypoxia, RSUME expression is induced, leading to an increase of HIF-1*α* SUMOylation, stabilization, and transcriptional activity. However, a recent study indicates that the hypoxia-induced HIF-1*α* SUMOylation targets this protein for degradation through the von Hippel-Lindau (VHL) protein-mediated ubiquitin proteasome pathway [97, 98]. The activation of signaling cascades also favors the crosstalk between SUMO and other PTMs. Phosphorylation regulates SUMO conjugation of multiple transcription factors through the PDSM motif [38] (Figure 3), including heat-shock factors (HSFs), myocyte enhancer factor 2 (MEF2), and oestrogen-related receptors (ERRs) *α* and *γ* [99–102]. This phosphorylation-dependent regulation of SUMOylation has been referred as a phospho-sumoyl switch [103]. Furthermore, lysine residues involved in SUMOylation are also targets of other PTMs, including ubiquitylation, acetylation, and methylation. For instance, SUMO conjugation can occur on the same lysine residue used to promote ubiquitylation of I*κ*B*α* resulting in a competition between these PTM [40]. However, SUMOylation and ubiquitylation do not necessarily compete with each other as, in some cases, SUMOylation acts as a recognition signal for an ubiquitin ligase [97]. The interplay between SUMOylation and acetylation has been observed in the regulation of proteins such as MEF2, histone, and hyper methylated in cancer 1 (HIC1) [104–107]. In the case of MEF2, the SUMOylation-acetylation switch is regulated by phosphorylation [105]. Altogether, these data demonstrate that multiple signaling cascades are regulated by SUMOylation with an intensive crosstalk between PTMs.

 The type of analysis developed here can be used to visualize individual and global processes regulated by SUMOylation. In this way, the study of SUMO-targets will not be isolated but integrated with the rest of the SUMO-regulated processes. Beyond the identification of molecular processes and signaling cascades, IPA can also be used for the identification of biomarkers of a given process or pathology where SUMOylation plays a critical role (Supplementary Table  6). In the future, this information could help us to identify pathologies, treat diseases, and predict responses to avoid treatments that will activate unwanted side effects. The number of available drugs that potentially affect SUMO regulated processes is not negligible so one can envisage the possibility to use them to tackle signaling cascades, molecular events and/or diseases where SUMOylation is critical (Supplementary Table  6). This approach could accelerate our understanding of the role of SUMOylation in many essential cellular events.

## 7. Concluding Remarks

SUMOylation just as other PTMs contributes to the regulation of multiple processes in the cell. To investigate the role of SUMO on the function of a given protein or pathway, the main approach considers the identification of the sites of modification or the sequences interacting with SUMOylated proteins. In contrast to ubiquitylation, SUMOylation sites can be predicted using one of the available algorithms published by several groups. However, those programs are not 100% reliable as they do not consider several aspects that regulate the SUMOylation of a protein. Here, we have analyzed all motifs present in human proteins reported in the PhosphoSitePlus (http://www.phosphosite.org/) that have been proven as SUMOylated using multiple approaches and found that most of the proteins contain the consensus [IVL]KxE. Before going through the identification of one substrate or pathway of interest, it is important to verify the public information available. There is not a single database that includes all published information of putative SUMO modified proteins identified by MS. However, the PhosphoSitePlus database includes SUMO sites that have been demonstrated by several groups using several methodologies. It is important to underline that while the lists of proteins identified using MS and other approaches can be counted by hundreds, the number of SUMOylation signatures identified from endogenous modified proteins remain low (no more than 150). All this information can be integrated in a rational manner to identify within a pathway, proteins that have been linked to SUMOylation. More importantly, this type of analysis can be used to identify biomarkers for a given process or disease and/or choose possible targets for therapeutic intervention (Supplementary Table  6). A long list of those targets has been used to develop drugs that can potentially be exploited to characterize processes or pathologies were protein regulation by SUMOylation is essential.

## Supplementary Material

Supplementary material include databases of putative and real sumoylated proteins (Table 1), overlapping of datasets of putative and real sumoylated proteins (Table 2), databases of sumoylated peptides (Table 3), molecular functions of putative and real sumoylated proteins (Table 4), canonical pathways of putative and real sumoylated proteins found using IPA (Table 5), and biomarkers and potential drug targets found in putative and real sumoylated proteins using IPA (Table 6).

## Figures and Tables

**Table 1:** SUMO/Sentrin specific proteases. SUSPs/SENPs implications and functions. Adapted from Wilkinson and Henley, 2010 [3].

Species	Name	Tissue expression	Localization	Preference	Processing	Deconjugation	Chain editing
*S. cerevisiae *	Upl1	NA	Nuclear periphery	NA	Yes	Yes	No
	Upl2	NA	Nucleoplasm	NA	No	No	Yes
Mammals	SENP1	Testes (high), pancreas, spleen, liver, ovaries, small intestine, thymus (low).	Nuclear pore and Nucleoplastic speckles	S1 > S2/3	Yes	Yes	No
	SENP2	ND	Nuclear pore	S2/3 > S1	Yes	Yes	No
	SENP3	ND	Nucleolus	S2/3	ND	Yes	No
	SENP5	ND	Nucleolus	S2/3	Yes	Yes	No
	SENP6	ND	Nucleoplasm	S2/3	No	No	Yes
	SENP7	Testes (high), pancreas, ovaries, colon, peripheral blood.	Nucleoplasm	S2/3	No	No	Yes
