# Supplementary material for: Strategies to Identify Recognition Signals and Targets of SUMOylation
Source: Biochem Res Int. 2012 Jul 1;2012:875148. doi: 10.1155/2012/875148 (PMC3395311; doi:10.1155/2012/875148)
Supplement: Supplementary file 1 — Supplementary material include databases of putative and real sumoylated proteins (Table 1), overlapping of datasets of putative and real sumoylated proteins (Table 2), databases of sumoylated peptides (Table 3), molecular functions of putative and real sumoylated proteins (Table 4), canonical pathways of putative and real sumoylated proteins found using IPA (Table 5), and biomarkers and potential drug targets found in putative and real sumoylated proteins using IPA (Table 6). [file 875148.f1.pdf]

Supplementary Legends

Supplementary Figure 1: Integrated view f the role of SUMO in the Glucocorticoid Receptor Signalling pathway. Ingenuity analysis of proteins that have been identified (in gray) in recent studies: PGK1, PKM2, ACSL3, PKLR, GAPDH, LDHB, ADH5, HK1, GPI, HK2, ENO1, ALDH3A2, DLAT, ALDH1A2, DLD, ALDOA, LDHA and ALDOC (Figure 4 and supplementary table 1) by mass spectrometry using His-6-SUMO-tagged.

Supplementary Figure 2: Integrated view f the role of SUMO in the Ubiquitin-Proteasome pathway. Ingenuity analysis of proteins that have been identified (in gray) in recent studies: USP14, ANAPC1, HSPA5, USP39, USO1, HSPA4, HSP90B1, HSP90AB1, PSMC6, BRCA1, UBB, DNAJC9, HSPH1, HSPA9, MDM2, HSPD1, PSMD3, HSPA8, ANAPC4, PSMC1, PSMD2, PSMD12, HSP90AA1, PSMD1, UBC, USP34, USP25, VHL and UBE2I (Figure 4 and supplementary table 1) by mass spectrometry using His-6-SUMO-tagged.

Supplementary table 1. Databases of putative and real sumoylated proteins used in this work.

Supplementary table 2. Overlapping of datasets of putative and real sumoylated proteins used in this work.

Supplementary table 3. Databases of sumoylated peptides used in this work.

Supplementary table 4. Molecular functions of putative and real sumoylated proteins found using IPA.

Supplementary table 5 Canonical pathways of putative and real sumoylated proteins found using IPA.

Supplementary table 6. Biomarkers and potential drug targets found in putative and real sumoylated proteins using IPA.

Proteins considered in all comparisons

blomsterMCP.txt not , Gallison.txt, matic.txt, phosphosite.txt:

293@,DDX3X,PRMT5,HNRPDL,NKRF,MAGEB2,CFLAR,PSMD3,HNRNPR,PRPF3,NARS,NRD1,SYNCRIP,EIF5B,H2AFY,CS,SLC25A12,RSL1D1,SRP72,MTA2,ALDH1A2,PRPF6,IKBKAP,SMC2,AHSA1,STAU1,LDHA,PNP,PGK1,HBZ,ALB,TFRC,ALDOA,ANXA1,TUBB4A,HIST1H2AB,HIST1H2AE,ATP1A1,SLC25A5,EIF2S1,RPLP0,ATP5B,ENO1,GPI,LDHB,ANXA2,TUBB,PFN1,HSP90AA1,HSP90AB1,SRPR,ASNS,HMBS,SNRNP70,VIM,RPSA,DLD,ALDOC,DLAT,HSPD1,HSPA5,HSPA8,ACADM,G6PD,ADH5,PABPC1,IMPDH2,CKB,EEF2,PDIA4,P4HA1,FDPS,PKM2,HSP90B1,DARS,RPS2,HIST1H1B,FAH,UBTF,TCP1,RPL7,V

CL,RPL17,HK1,EIF2AK2,VDAC1,GART,PAICS,WARS,RPS3,AHCY,MCM3,ATP5A1,MSN,U2AF2,APEX1,TKT,EEF1D,RPL12,PDIA3,NMT1,PKLR,SERPINB1,SDHA,RRM2,ATIC,PYCR1,MCM4,MCM5,HSPA4,RPL22,FUS,ATP5C1,CPOX,PPP1CC,TALDO1,RBMX,HSPA9,RPL3,FEN1,CCT6A,RPL13A,STAT3,MDH1,MDH2,HADHA,GARS,STAT5A,LRPPRC,MSH2,VDAC2,RPL5,CAPZB,ARCN1,CCT5,CCT3,TUFM,AARS,SARS,MCM2,GMPS,RPL14,CCT8,CCT4,MSH6,HK2,CAPZA1,BLVRA,ACLY,YARS,USP14,VCP,NAP1L1,MARS,EIF4A1,RPS3A,ATP6V0D1,RPS7,PPP1CA,PPP1CB,PSMC1,RPS8,RPS16,RPS18,PSMC6,RPL7A,RPS6,HIST1H2BC,HIST1H2BE,HIST1H2BF,HIST1H2BI,HIST1H2BJ,RAN,PPIA,AP2B1,GNAI1,GNB2L1,ACTC1,EEF1A1,TUBA1B,TUBB4B,CCT2,MPP1,CAP1,EXOSC10,PLCB3,RPL18A,RPL6,AKR1C1,AKR1C3,PPAT,PRDX1,RPL18,DHX9,ILF2,ILF3,TRAP1,HNRNPA0,PSMD2,PABPC4,NACA,TUBB2A,HNRNPD,BOP1,FLNC,RBM39,MCM6,SMC1A,RRP1B,GANAB,CHD4,SART3,EFTUD2,KARS,PPA1,NONO,PCBP1,PCBP2,SF3B3,DDB1,FSCN1,NDUFA9,IMMT,HNRNPUL2,TIMM50,LARP7,USP39,CDCA8,RRP12,PRPF8,DARS2,TUBA1A,SND1,DHX30,FERMT3,DDX42,KIAA1967,NUP93,LRRC47,ABCF1,PRPF31,DNAJC9,DDX1,PSMF1,HSPH1,MYO18A,GCN1L1,DHX38,NUP205,ANP32B,DDX17,UPF1,DDX27,RBM14,SRPK1,PSMD1,POP1,PHB2,CCT7,EBNA1BP2,PRMT1,DDX50,WDR77,TUBB6,DDX23,UTP14A,ACAT2,SPATA16,GTPBP4,WDR33,XRN2,GHITM,FN3K,RNPEP,DDX21,NANS,FARSB,SMC4,ATAD3A,RBM28,IGF2BP1,RCC2,LARS,DHCR7,SRP68,RALY,CORO1C,PRPF19,RPL26L1,VDAC3,FARSA,DRG1,UTP18,PSAT1

Gallison.txt not , blomsterMCP.txt, matic.txt, phosphosite.txt:

306@,BTBD11,PGRMC1,PIK3C2A,NOP56,AP3D1,CHD1,CHD2,SLC27A2,ZNF263,ZNF609,ZNF646,ZBTB5,ERC2,AXIN1,YY2,DHX15,DAPK3,TBX1,PLRG1,NDUFB3,URB1,ZIC3,CCNT2,USO1,ABCB7,PRPF40A,MAF,ERLIN1,NUP155,ZMPSTE24,ARL6IP5,ZNF202,LETM1,TNKS,ZIC2,SGPL1,WIZ,DLGAP3,ONECUT2,HAND1,CA2,KRT14,KRT1,RPN1,ARG1,EPHX1,SLC3A2,CYC1,KRT19,H2AFZ,KRT10,KRT13,JUP,DSP,HIST1H1D,ATP2A2,ZNF37A,XBP1,SON,POU3F3,POU3F2,RFX1,ATP2B4,ABCD3,HOXA9,OTX1,KRT9,DDOST,TMPO,RPL27A,STT3A,CAMLG,SSR4,BCAP31,ATP1B3,BRPF1,FOXG1,MAZ,TMEM33,NUP107,SEC61B,RAB2A,RPL15,RAB1A,UBA52,TRA2B,SEC11A,NOP14,REEP5,SPTBN1,DSG1,CREB5,KRT17,DLX2,BAX,DSC1,RBBP4,NCBP1,NUP160,BPTF,TP53BP1,LMAN2,FOXF2,NNT,SLC39A6,PRPF4B,STIM1,TRA2A,DYRK1A,SPTAN1,PKP1,MLEC,KRT81,ITPR3,TRIP12,LBR,ARID5B,NOLC1,KRT72,NCAPD2,PWP2,KRT31,KIAA0020,SF3B4,TSC22D1,NSDHL,CPEB4,AMOT,GLE1,ZNF326,NOM1,FLG2,FAM76B,MIA3,DNTTIP2,NUP188,BEND3,KPRP,RIF1,CACHD1,ARID2,INTS3,KIAA1429,IQSEC1,SUMO4,DHRS7B,TMEM214,TAF2,ZC3H14,TMEM205,PTPLB,RNF111,USP34,HIST2H3D,HIST2H3C,HIST2H3A,CBLL1,LPCAT2,HIST3H2A,NUFIP2,MTDH,CAND1,HRNR,PHF6,ELMOD2,MLL5,ZNF687,KRT78,STAG2,JAGN1,C4orf32,GDPD1,UBAC2,SCCPDH,LEMD2,SLC30A7,LPCAT1,ARID1B,SMARCC2,PMFBP1,RDH11,STT3B,HM13,NUP210,NOC3L,PHIP,SYNE2,AUTS2,BRI3BP,SLC39A7,CENPI,TNPO1,NKD2,SMARCE1,NCLN,CIRH1A,LRRC59,RBM33,TMX3,ZNF462,IPO9,PPP1R10,CLCC1,TMEM209,SPEN,RNF2,CDX2,ATXN2,HIST1H2BN,ADPGK,ESYT1,TMEM43,TMEM48,C1orf35,NOC4L,NUP85,FANCD2,KIAA1715,BRD8,ANAPC1,TMX1,ZNF644,ZNF768,NOL6,NOL11,FBRS,XPO5,PREB,BRD7,OSTC,KRT82,BMP2K,PDS5B,SACM1L,FANCI,SLTM,SMPD4,TEX10,CDK12,TECR,CALML5,PTPLAD1,TMEM111,H2AFY2,SENP1,ZBTB4,ZNF319,RERE,STX18,TFIP11,SEL1L,SEC63,SUN2,CCNL1,GPATCH8,ZNF777,ZNF608,SLC39A10,YEATS2,TMCO1,SSR3,TIMELESS,FOXJ3,SMC3,LRCH1,LEMD3,TNPO3,MAFB,GTF3C3,AUP1,DNMT3A,TUBB2B,DGKE,HIST1H2AG,HIST1H2AI,HIST1H2AK,HIST1H2AL,HIST1H2AM,WDR75,VAPA,S100A9,PDS5A,WDR3,CDIPT,HSD17B12,YWHAZ,PDS5B,UBC,UBB,UBA52,RPS27A,GATA6,RDH11,ZNF777,VAPB,DCD,SRPRB,TRA2A,KTN1,RAB1A,EMD,ALDH3A2,CYCS,ACSL3,ORC5,TPR

blomsterMCP.txt, Gallison.txt not , matic.txt, phosphosite.txt:
57@,PES1,XPO1,ACTN4,SMARCA5,DKC1,SNRNP200,GAPDH,HSPA1A,HSPA1B,SLC25A6,HNRNPL,HIST1H1C,NCL,FBL,HNRNPA2B1,SFPQ,RPL13,PTBP1,HNRNPH3,HIST1H2BB,MYH9,MYH10,IARS,MATR3,FASN,COPA,CSE1L,RAB10,HIST1H4A,HIST1H4B,HIST1H4C,HIST1H4D,HIST1H4E,HIST1H4F,HIST1H4H,HIST2H4A,HIST2H4B,HIST1H4I,HIST1H4J,HIST1H4K,HIST1H4L,HIST4H4,PRKDC,SLC25A3,CLTC,SSRP1,MDC1,PDCD11,BMS1,KPNB1,H2AFV,CYFIP1,FTSJ3,MYBBP1A,DDX18,BAZ1B,TLN1

matic.txt not , blomsterMCP.txt, Gallison.txt, phosphosite.txt: 5@,SAP18,SNRPD2,AHNAK,ZBTB2,SLK

blomsterMCP.txt, matic.txt not , Gallison.txt, phosphosite.txt: 0@

Gallison.txt, matic.txt not , blomsterMCP.txt, phosphosite.txt: 2@,CANX,BRD4

blomsterMCP.txt, Gallison.txt, matic.txt not , phosphosite.txt: 0@

phosphosite.txt not , blomsterMCP.txt, Gallison.txt, matic.txt:
209@,KCNK1,PSMD12,ARNTL,DNM1L,NR5A2,RGPD8,TCERG1,MDM4,ATXN7,TP73,MAFG,ZEB2,TBL1X,LECT1,ZBTB7A,KLF8,NFATC1,KRT75,DHFR,SOD1,FOS,KRT6A,ESR1,NR3C1,KRT6B,TP53,TYMS,APP,JUN,SSB,KRT8,RB1,PGR,SP1,IGF1R,NR3C2,KRT7,ANXA5,MYB,AR,RARA,IRF1,SRF,SKIL,IRF2,ETS1,GATA1,CREB1,ATF7,CAPN2,CEBPB,PTPN1,ELK1,WT1,RXRA,KCNA5,SP100,CDK2,YY1,NFKBIA,PDE4A,ARID4A,KDM5A,SHMT1,RORA,CBS,SOX6,ZNF76,SREBF1,PPARG,SNCA,BRCA1,VHL,ETV5,ETV6,ELK3,CASP2,HTT,SLC1A2,ETV4,RAD52,NR4A2,YAP1,NRIP1,KRT6C,MAPKAPK2,CEBPD,GSK3B,PAPOLA,CLCN7,HDGF,BLM,ATXN1,NRL,NR1H2,HDAC4,SOX10,KLF3,FOXL2,UBE2K,ESRRG,UBE2I,HMGN5,HSF1,NFKB2,MDM2,FLI1,KRT76,SATB1,POU5F1,MEF2A,SP3,CENPC1,LMNB2,HSF2,ZBTB16,MEF2C,PPARA,EP300,SREBF2,NR1H3,MAP3K1,NR5A1,CTBP1,XRCC4,SMAD4,HDAC1,TDG,MORC3,IKBKE,WRN,HIC1,MEF2D,POLD3,TAF5,NCOA2,CEBPE,NCOA1,TAF12,NFE2,HIF1A,ZXDC,GLYR1,PLAG1,RGPD4,GATAD2A,AHNAK2,ZFPM1,CKAP2L,MYOCD,PIAS4,NIN,SNIP1,GATAD2B,MAML1,TFAP2C,PROX1,KHSRP,KAT5,MKL1,FUBP1,SIRT1,FYTTD1,CDK5RAP2,PARK7,FMO2,ELF4,RGPD5,RGPD6,EPAS1,ARID3A,BACH2,TBL1XR1,SAP130,HIPK2,ZFP106,TP63,GCM1,DYRK4,PDGFC,TOPORS,SALL1,NHP2,ZNF234,ZNF226,HSFX1,MRC2,UBA2,ZMYM2,DAXX,IFT172,TRPS1,USP25,XPO7,ANAPC4,ZNF221,CPSF3,RCOR1,HSF4,PLAGL2,SCAF8,SATB2,PA2G4,RUVBL2,RUVBL1,NOP58,TBX22,IKBKG,NCOA3

blomsterMCP.txt, phosphosite.txt not , Gallison.txt, matic.txt:
9@,NPM1,EPRS,PCNA,DDX5,ACADVL,HNRNPA3,HNRNPK,XPC,SAFB

Gallison.txt, phosphosite.txt not , blomsterMCP.txt, matic.txt:
17@,CBX4,KRT3,KRT2,CTCF,HNRNPF,SUMO3,ZNF146,KRT79,MAFA,ATXN2L,CHD8,KRT84,MBD1,ZNF148,ZNF711,XRCC5,DYNC1H1

blomsterMCP.txt, Gallison.txt, phosphosite.txt not , matic.txt:
7@,HNRNPA1,TOP2A,LMNB1,DNMT1,HNRNPH1,HNRNPU,TOP2B

matic.txt, phosphosite.txt not , blomsterMCP.txt, Gallison.txt:
32@,TRIM24,CD3EAP,MYO1B,FOSL2,MAP4,STAT1,VASP,ADAR,KHDRBS1,RLF,NAB1,IFI16,ZNF800,ARID4B,ZMYM1,PTRF,CCAR1,RBM12B,FNBP4,ZBTB38,ZNF280C,KDM2B,GEMIN5,RREB1,SYMPK,ZBTB9,THOC1,ERBB2IP,HNRNPUL1,ZNF295,TRIM33,ZBTB1

blomsterMCP.txt, matic.txt, phosphosite.txt not , Gallison.txt:
6@,SART1,LMNA,PARP1,TOP1,ACTB,SAFB2

Gallison.txt, matic.txt, phosphosite.txt not , blomsterMCP.txt:
21@,KRT5,PML,RANGAP1,YLPM1,RBM25,RANBP2,SUMO2,SUMO1,ACTG1,GTF2I,TRIM28,TCOF1,ZMYM4,PBRM1,RSF1,PNN,BCLAF1,ACIN1,ZMYND8,ZNF451,GTF2I
intersect all: 6@,SF3B1,HNRNPC,FLNA,MKI67,HNRNPM,NUMA1

93% of all proteins reported by Matic et al have been reported in at least one other study.

The biggest pairwise overlap of SUMOylated proteins was found between Matic and phosphosite with a overlap of 20.5%.

All pair vise comparisons:

blomsterMCP.txt vs Gallison.txt	307 71 346
blomsterMCP.txt vs matic.txt	365 13 59
blomsterMCP.txt vs phosphosite.txt	350 28 278
Gallison.txt vs matic.txt	389 28 44
Gallison.txt vs phosphosite.txt	367 50 256
matic.txt vs phosphosite.txt	8 64 242
