# Supplementary figures and images for: Strategies to Identify Recognition Signals and Targets of SUMOylation
Source: Biochem Res Int. 2012 Jul 1;2012:875148. doi: 10.1155/2012/875148 (PMC3395311; doi:10.1155/2012/875148)

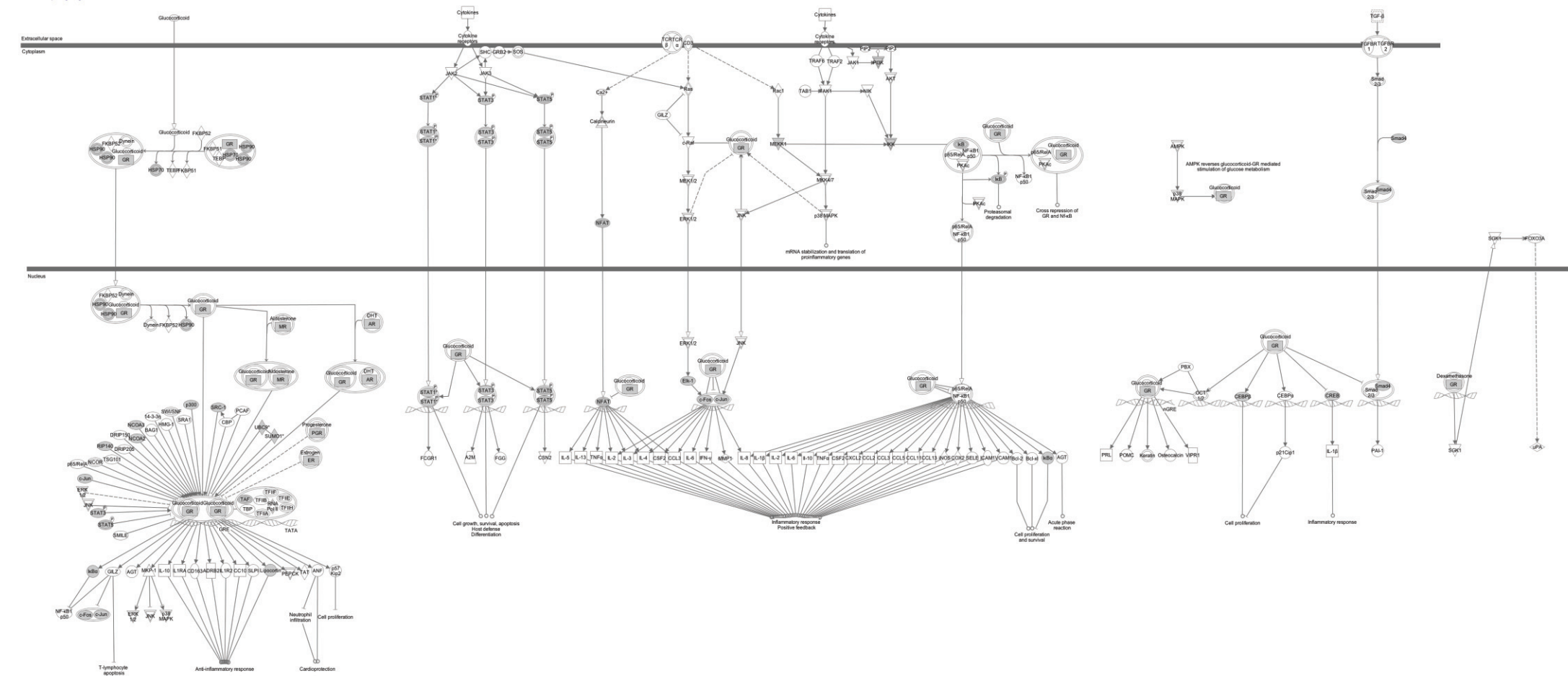

Supplement: Supplementary file 2 [file 875148.f2.pdf]

### Protein Ubiquitination Pathway

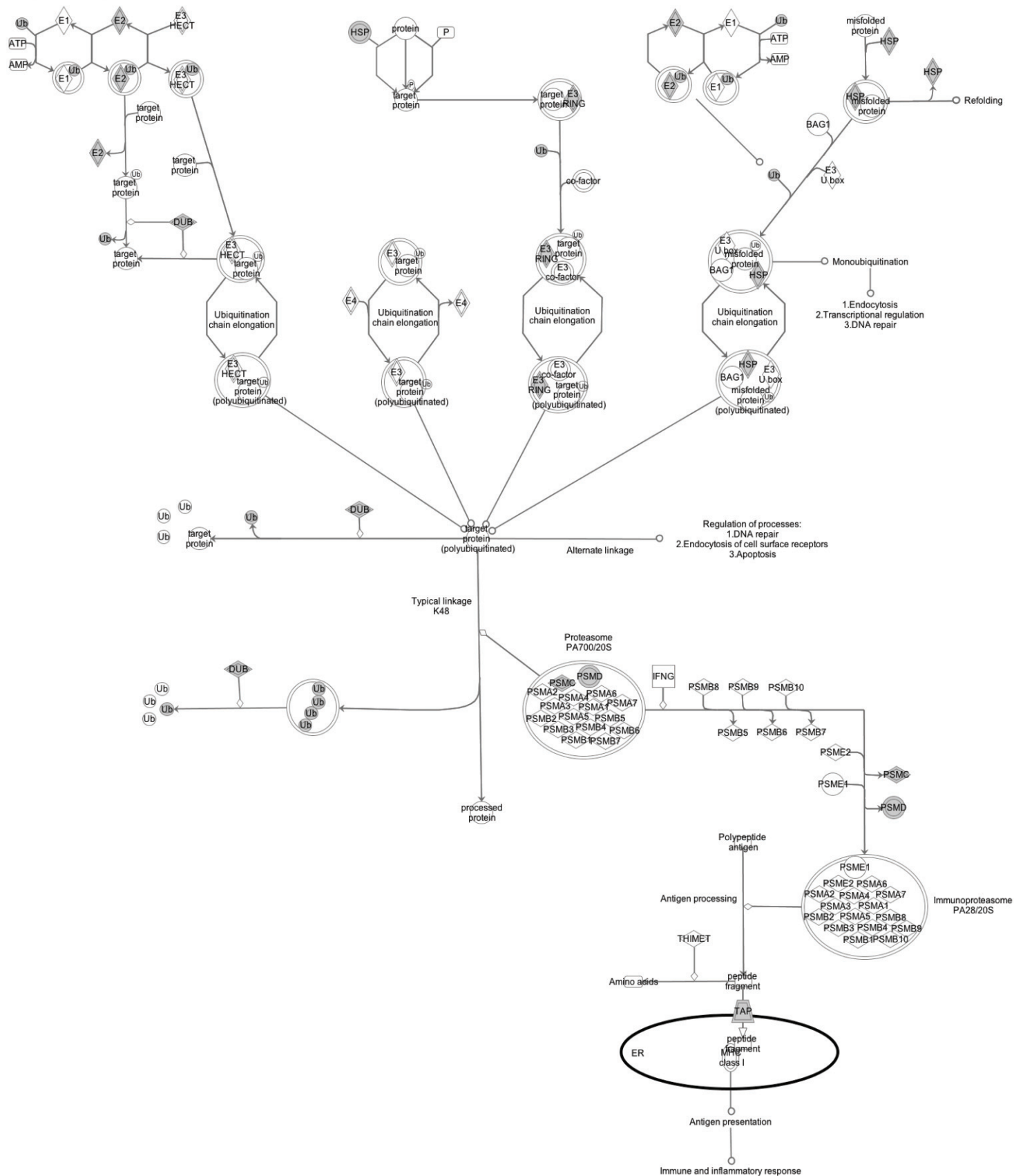

Supplement: Supplementary file 3 [file 875148.f3.pdf]
